# Mucoepidermoid Carcinoma of the Lung: A Case Report and Literature Review

**DOI:** 10.1155/2013/625243

**Published:** 2013-11-04

**Authors:** S. Alsidawi, J. C. Morris, K. A. Wikenheiser-Brokamp, S. L. Starnes, N. A. Karim

**Affiliations:** ^1^Department of Internal Medicine, University of Cincinnati, Cincinnati, OH 45267, USA; ^2^Division of Hematology-Oncology, Department of Medicine, University of Cincinnati, Cincinnati, OH 45267, USA; ^3^Pathology and Laboratory Medicine, Cincinnati Children's Hospital Medical Center and University of Cincinnati College of Medicine, Cincinnati, OH 45267, USA; ^4^Division of Thoracic Surgery, Department of Surgery, University of Cincinnati, Cincinnati, OH 45267, USA

## Abstract

*Introduction*. Mucoepidermoid carcinoma (MEC) of the lung is a rare form of lung cancer that is classified into low grade and high grade based on histological features. Surgical resection is the primary treatment for low-grade MEC with excellent outcomes, while high-grade MEC is a more aggressive form of malignancy. *Clinical Case*. We report a case of a 46-year-old woman who presented with dyspnea on exertion. Imaging studies revealed a mass involving the right upper lobe bronchus. Bronchoscopy, surgical resection, and pathological examination revealed a low-grade MEC with tumor-free margins. No adjuvant treatment was given. *Discussion*. Primary pulmonary MEC is a rare type of lung cancer with only few reported cases. This patient illustrates a typical presentation for low-grade MEC wherein surgical resection is considered curative. In contrast, high-grade MEC is a more aggressive malignancy with a poorer outcome. The role of targeted therapy directed against EGFR or a novel CRTC1-MAML2 fusion protein expressed in some high-grade tumors is yet to be determined.

## 1. Background

Mucoepidermoid carcinoma (MEC) is a rare tumor of the lung that accounts for 0.1 to 0.2% of all pulmonary tumors [1]. MECs most often arise from the parotid or submandibular salivary glands. Most pulmonary MECs arise in the proximal bronchi. Histologically, MEC is characterized by a combination of mucus-secreting, squamous, and intermediate cell types. Low-grade MECs are comprised predominantly of glandular elements and mucin-secreting cells, while high-grade MEC consists largely of sheets or nests of squamoid and intermediate cells intermixed with smaller populations of mucus-secreting cells. Molecular techniques in salivary and pulmonary MECs have shown a particular chromosome (11; 19) translocation generating a novel CRTC1-MAML2 fusion protein [2, 3]. This novel protein acts as a transcription factor functioning in cell growth regulatory pathways. It contributes to tumor development by the disruption of normal cell cycle control and cellular differentiation.

Pulmonary MEC patients typically present with symptoms related to bronchial obstruction and atelectasis, such as cough, hemoptysis, wheezing, and postobstructive pneumonia [4]. The prognosis of localized low-grade disease is excellent, with very good 5- and 10-year survival rates reported in various case series. Locally advanced high-grade disease has a much more guarded prognosis with the majority of patients succumbing to their disease.

## 2. Case Presentation

**Figure 1:**
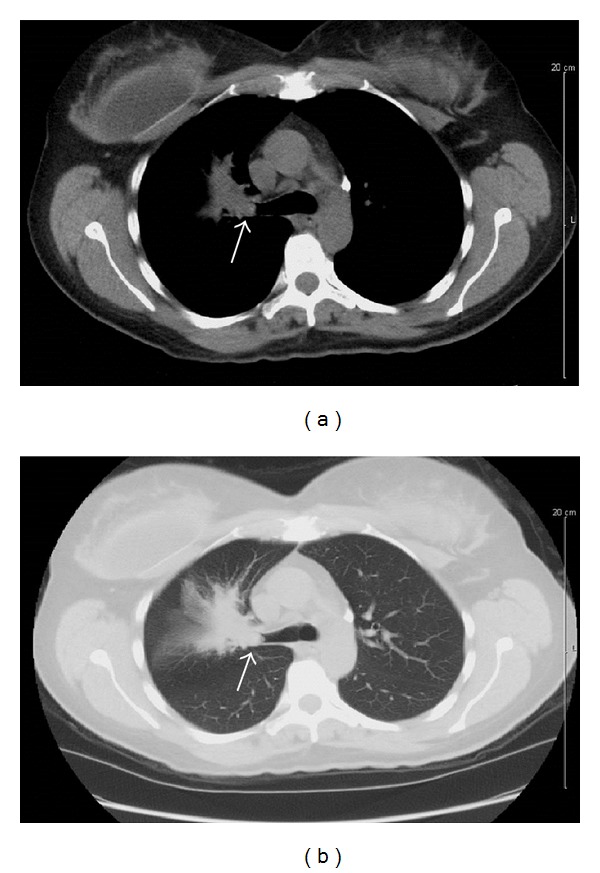
Computerized tomography (CT) of the chest showing a mass (arrow) measuring 1.5–2 cm obstructing the right upper bronchus with associated atelectasis. No evidence of metastatic disease is seen. (a) Mediastinal window. (b) Lung window.

**Figure 2:**
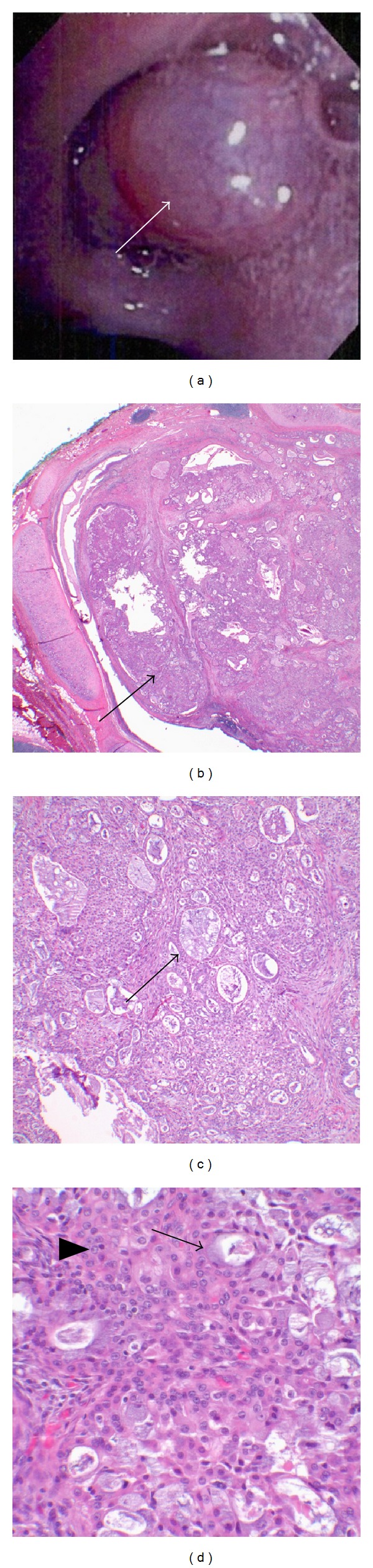
(a) Bronchoscopic image showing a smooth, well-circumscribed endobronchial tumor (arrow) at the orifice of the right upper lobe bronchus. (b) Low power (20x) microscopic image showing a polypoid endobronchial mass extending into the bronchial lumen (arrow) and superficially invading the submucosa. (c) Tumor is comprised of glands, tubules, and cysts containing mucin (arrow) separated by a fibrous stroma (100x). (d) Higher power (400x) microscopic image showing a mixture of mucin-secreting cells (arrow) admixed with sheets of squamoid and intermediate cells (arrowhead) intimately admixed with the glandular component. The cells lack significant mitotic activity, nuclear pleomorphism, and cellular necrosis characteristic of a low-grade mucoepidermoid carcinoma.

A 46-year-old Caucasian female without a significant past medical history presented with complaints of several months of increasing dyspnea on exertion. She was an avid cyclist; however, her dyspnea prevented her from performing any form of exercise for the previous several months. She also reported increased fatigue, dry cough, and occasional wheezing. The patient had a 2.25 pack-year (0.25 packs/day for 9 years) smoking history before she quit twenty years prior to presentation. A chest radiograph revealed right upper lobe collapse. Computerized tomography (CT) of the chest showed a mass involving the right upper lobe bronchus with associated atelectasis (Figures 1(a) and 1(b)). Bronchoscopy was performed and demonstrated a smooth, well-circumscribed tumor at the right upper lobe orifice (Figure 2(a)) that was presumed to be a carcinoid; however, a biopsy of the mass was nondiagnostic. A decision was made to proceed with surgery. The patient underwent a thoracotomy with right upper lobectomy with sleeve resection and mediastinal lymph node dissection. Pathological examination revealed a 1.5 cm tan-yellow, well-circumscribed mass within the bronchial lumen that did not grossly invade into the surrounding lung parenchyma. Microscopic examination revealed a low-grade mucoepidermoid carcinoma (Figures 2(b)–2(d)). All resection margins were negative for tumor involvement, and the lymph nodes were free of metastatic disease. The patient tolerated surgery well and postoperatively reported improvement of her symptoms. No adjuvant treatment was recommended and the patient continues to follow up with surveillance imaging.

## 3. Discussion

The World Health Organization (WHO) classifies pulmonary MECs as “salivary gland type” tumors along with pulmonary adenoid cystic carcinomas and epimyoepithelial lung carcinomas [5]. There are only a few cases of primary pulmonary MEC reported, most occurring in younger age groups as compared to the other more common types of lung cancer [6]. Histologically, MEC is comprised of a mixture of different cell types including mucin-secreting glandular cells, squamous cells, and intermediate cells. Low-grade MEC is distinguished from high-grade MEC based on the lack of cytological atypia including nuclear pleomorphism and absence of significant mitotic activity and cellular necrosis. Histological grade is an important prognostic indicator, with high-grade MECs demonstrating a greater risk for metastases, tumor recurrence, and death [7]. Heitmiller et al. reported their experience of 18 patients with MEC [4]. The patients' tumors were classified into low-grade or high-grade carcinomas based on the degree of mitotic activity, presence of necrosis, and nuclear pleomorphism. All patients with low-grade tumors were alive at a mean followup of 4.7 years, while all patients with high-grade tumors died within 16 months. Of note, some of the high-grade tumors in this study were not amenable to surgical resection at the time of diagnosis given the local extension of their disease. There is data to suggest that expression of matrix metalloproteinases is less robust in low-grade compared to high-grade MECs, and this difference in expression, at least in part, may explain the less aggressive behavior of low-grade MECs [8].

While surgical resection remains the standard therapy for patients with pulmonary MEC [9], different operative approaches have been used. Recently, video-assisted thorascopic surgery (VATS) has become the most frequently used technique for resection of MECs. Breyer et al. treated five patients with MEC with different surgical approaches including thoracotomy with conventional lobectomy, sleeve lobectomy, and lobectomy, with bronchoplastic closure. No differences in outcome were observed among the various surgical modalities [10]. The goal of surgery is to obtain a complete resection with negative surgical margins. Radiation therapy has been used to treat high-grade MECs with an inconclusive effect on patient survival. El Mezni et al. reported their experience in 10 patients with MEC occurring at a mean age of 43.9 years including five low-grade and five high-grade tumors. All 10 patients underwent surgery (lobectomy or pneumonectomy), and two patients received postoperative radiation therapy. Three patients died of disease. Two of these patients had high-grade MEC and one had a low-grade lesion. The remaining seven patients were alive without evidence of recurrence [11].

Patients with low-grade MECs have a generally excellent prognosis with a five-year survival rate approaching 95%. In this population, adjuvant therapy is not indicated. In contrast, high-grade MECs carry a much poorer prognosis [12, 13].

Leonardi et al. followed seven patients with MEC, six low-grade and one high-grade lesions, that underwent different surgical approaches as a primary treatment. The average survival rate for low-grade MEC was 12.8 years, while the patient with the high-grade tumor died 28 months after diagnosis despite two attempts at surgical resection and local radiation treatment [12].

Our patient had a typical presentation for a low-grade MEC, a single centrally located well-circumscribed endobronchial tumor without evidence of locoregional or distant metastasis. The tumor was resected by sleeve lobectomy in combination with mediastinal lymph node dissection. Histopathological findings were diagnostic of a low-grade MEC with a confirmed complete tumor resection with negative surgical margins and no evidence of metastatic spread to lymph nodes. Based on the experience of multiple groups in treating low-grade MEC [4, 7, 9–11, 13], surgical treatment is curative in this group of patients. No studies support the use of adjuvant chemotherapy, targeted therapy, or radiation therapy for low-grade pulmonary MECs given the excellent survival rates achieved with surgery alone.

Due to the relatively small number of reported cases of high-grade pulmonary MECs, there is no consensus on adjuvant treatment for this group of patients. Given that the epidermal growth factor receptor (EGFR) is frequently overexpressed in MECs of salivary gland origin, Han et al. tested for EGFR mutations in pulmonary MEC specimens [14]. EGFR mutations were found in two of five, with both mutations resulting in a nonconservative substitution of leucine for arginine at position 858 (L858R). Two of the three cases also exhibited high levels of EGFR polysomy by fluorescence in situ hybridization (FISH) and EGFR overexpression by immunohistochemistry. In another study, Yu et al. identified a heterozygous exon 21 leucine to glutamine mutation (L861Q) in five of twenty MEC tumors collected over nine years [15]. No deletions in exon 19 or exon 21, as typically seen in non-small cell lung cancer, were detected. Han et al. tested the effectiveness of tyrosine kinase inhibitor (TKI) therapy by treating a patient with recurrent metastatic MEC who progressed on multiple chemotherapy regimens with the EGFR-specific TKI gefitinib. Treatment resulted in radiographic evidence of a partial response in this patient [14]. Rossi et al. also administered gefitinib to a patient with metastatic high-grade MEC and observed regression of subcutaneous metastases and stabilization of pulmonary disease that was progressing on conventional chemotherapy [16]. Interestingly, no EGFR mutations were detected in the five pulmonary MEC tumors examined in the previous study. Additionally, Macarenco et al. found no EGFR mutations in twelve MEC tumors studied [17]. Lee et al. reported a patient with aggressive high-grade MEC treated with the TKI erlotinib who also showed radiographic evidence of partial response [18]. Despite these case reports, the role of TKI therapy in metastatic MECs remains unclear, especially given the finding that no activating EGFR mutations were detected in the tumors of patients who reportedly responded to the TKI therapy. Interestingly, studies on different lung cancer cell lines suggested that NCI-H292, a pulmonary MEC cell line that has wild-type EGFR, is more sensitive to gefitinib than other wild-type EGFR non-small cell lung cancer cell lines [19]. O'Neill analyzed the data from multiple studies and raised the interesting question that different ethnic populations may have different EGFR mutations in their pulmonary MEC tumors [20]. Per review of the literature, 48 pulmonary MEC tumors were tested for EGFR mutations, and nine tumors (19%) tested positive for mutations including two reports of L858R mutations, five L861Q mutations, one I760I mutation, and one exon 19 deletion. Of note, all the EGFR mutations were detected in the Asian population. Whether treatment with TKIs improves outcome in these patients remains unclear. Interestingly, Wong et al. detected an echinoderm microtubule-like protein-4-anaplastic lymphoma kinase-1 (EML4-ALK) translocation in two out of twelve pulmonary MEC tumors tested for this fusion gene [21].

Other gene rearrangements that might serve as potential novel targets are under investigation. It has been discovered that pulmonary MECs may harbor a t(11; 19) translocation with an associated novel fusion oncogene (CRTC1-MAML2) [22–24]. Fusion oncogenes have been successfully targeted for treatment in other malignancies such as chronic myelocytic leukemia (CML) leading to changes in therapeutic approaches. CRTC1-MAML2 translocations have been well studied in MECs of the salivary glands and were found to occur in 60–70% of cases [2, 23, 25]. Recent reports also demonstrate t(11; 19) translocations in lung MECs [26–28]. The fusion of exon 1 of the CTRC1 gene on chromosome 19p13 with exons 2–5 of the MAML2 gene on chromosome 11q21 generates a novel fusion oncogene, CRTC1-MAML2, that acts as a transcription factor altering Notch and CREB regulatory pathways, leading to disruption of normal cellular growth and differentiation that contributes to tumor development [29, 30].

## 4. Conclusions

Primary pulmonary MEC represents a rare type of lung cancer. Patients with low-grade MECs, like the patient presented in this report, generally have a good prognosis after primary surgical resection. Adjuvant treatment is not indicated for these patients. In contrast, high-grade pulmonary MECs are aggressive malignancies with most patients succumbing to the disease. The role of targeted therapy directed against EGFR or a novel CRTC1-MAML2 fusion protein expressed in some tumors is yet to be determined. Molecular profiling of these rare tumors may identify additional “druggable” targets.
